# Subsequent primary neoplasms among bone sarcoma survivors; increased risks remain after 30 years of follow-up and in the latest treatment era, a nationwide population-based study

**DOI:** 10.1038/s41416-020-0748-3

**Published:** 2020-02-18

**Authors:** Asle Charles Hesla, Andrea Discacciati, Panagiotis Tsagkozis, Karin E. Smedby

**Affiliations:** 1 Department of Molecular Medicine and Surgery (MMK), K1, Orthopaedics, Karolinska Universitetssjukhuset, 17176 Solna, Sweden; 2 Department of Medical Epidemiology and Biostatistics (MEB), C8, Biostatistics, Box 210 171 77, Stockholm, Sweden; 3 Department of Medicine, Solna (MedS), K2, Group K Ekström Smedby, Eugeniahemmet, T2, Karolinska Universitetssjukhuset, Solna, 171 76 Stockholm Sweden

**Keywords:** Risk factors, Sarcoma

## Abstract

**Background:**

The long-term risks and time trends of subsequent primary neoplasms (SPNs) among Ewing (ES) and osteosarcoma (OS) survivors are not fully understood.

**Methods:**

We performed a nationwide study of all ES and OS patients identified in the Swedish Cancer Registry from 1958 to 2015 with up to 58 years of follow-up. The risk of SPN was compared with that of the general population using standardised incidence ratios (SIRs) and absolute excess risks (AERs).

**Results:**

One hundred and fifteen SPNs were diagnosed among 1779 patients with ES or OS, yielding an overall SIR of 2.3 (95% confidence interval (CI), 1.9–2.7). The risk remained significantly increased in the latest treatment era (SIR_2000-2015_ 2.0; 95% CI, 1.1–3.5). The highest absolute excess risks (AER) was due to breast cancer (AER 15.2/10,000 person-years; 95% CI, 5.0–29.8) followed by female genital malignancies (AER 9.5/10,000 person-years; 95% CI, 2.4–21.5). The excess breast cancer risk among ES survivors was noted also after 30 years of follow-up with 127 extra breast cancers/10,000 person-years (95% CI, 6.6–419).

**Conclusions:**

Breast- and female genital malignancies contribute most to the excess risk of SPN among ES and OS survivors. Importantly, excess risks did not decline over calendar time or long-term follow-up.

## Background

Compared with the general population the increased risk for subsequent primary neoplasms (SPNs) in childhood cancer survivors has been clearly demonstrated in large studies in the U.S. and Europe.^1–5^ The Childhood Cancer Survivor Study (CCSS), which included patients treated between 1970 and 1999, observed a decreased risk for SPNs among childhood cancer survivors treated later in the study period. The lower risk was clearly associated with a reduction in the use of radiotherapy (RT) and radiation dose.^2,6^ Heritable retinoblastoma, Hodgkin lymphoma and bone sarcoma are the three primary childhood malignancies with the highest excess risk for SPNs.^2–4,6^ The increased risk can be attributed to genetic-, disease- or treatment related factors. Heritable retinoblastoma survivors are at increased risk due to germline inactivation of the retinoblastoma gene.^7^ Hodgkin lymphoma survivors have an increased treatment related cancer risk mainly driven by RT, but also by treatment with alkylating agents.^8,9^ Osteosarcoma (OS) and Ewing sarcoma (ES), the two most common childhood bone sarcomas, may have different aetiologies contributing to the excess cancer risk, yet they are commonly reported as one entity.^1,3^ Treatment with high dose multi-agent chemotherapy for OS and ES has hardly changed since the 1970s; however, only ES patients commonly receive RT, perhaps the most significant driver of excess cancer risk. Importantly, if a lower risk in recent treatment eras can be confirmed it may reflect a positive effect of a change in primary treatment such as decreased use of RT. Whether the risk persists with extended follow-up has important implications for recommendations on how long bone sarcoma survivors should be followed. Furthermore, as bone sarcoma survivors reach the age at which the cancer incidence in the general population increases significantly, it is important to identify which neoplasms contribute most to the excess risk. The aims of this nationwide population-based study were to; (1) estimate the risk of SPNs among ES and OS survivors; (2) investigate whether the absolute excess risk persists with long-term follow-up; (3) identify which subsequent neoplasms that contribute most to the excess risk; and (4) analyse the risk of SPNs among patients treated during more recent treatment eras.

## Methods

### Population and study design

This was a nationwide population-based register study. The cohort consisted of all ES and OS patients identified in the Swedish Cancer Registry during the period 1 January 1958–31 December 2015. All cancers occurring after the primary bone sarcoma diagnosis were included as outcomes in the analysis. The ES and OS patients were identified through International Classification of Diseases, 7th edition (ICD-7) codes in the Swedish Cancer Registry. Reporting with newer versions of the International Classification of Diseases (ICD-9 and ICD-10) was automatically converted to ICD-7 codes. The register includes data corresponding to a specific ICD-7 and morphology code of all new malignant tumours diagnosed in Sweden since 1958. Acceptance of a new cancer to the registry in a patient with an already known cancer follows the rules of the International Association of Cancer Registries (IACR).^10^ In addition, histologically benign tumours of the central nervous system (CNS) are also included in the register. All tumours of the lower urinary tract with cellular atypia, including papilloma of the bladder, are included in the register because of the difficulties in classifying the malignant potential of bladder tumours.^1,3^ Basal cell skin carcinoma is not included in the register. Reporting from multiple sources is required and the completeness of the register is over 96%.^11,12^ The methods of data collection in the register have been described elsewhere.^12^

The ICD-7 codes we used to identify the ES or OS cases by histology and anatomical location were: central location (196.2-196.3, 196.6); extremity (196.4, 196.5, 196.7, 196.8); unknown primary skeletal location (96.9); and soft tissue origin (197.1–3, 197.7). The morphological tumour type was ascertained from code 92603 (ES) or 91803 (OS) using the ICD-O WHO (World Health Organization) morphological classification of cancers, which has been used in Sweden since 1993.^13^ Before 1993 tumour morphology and tumour behaviour were coded according to the World Health Organization C24 code.^14^

### Subsequent primary neoplasms

Time at risk for a SPN started at the time of the first diagnosis of ES or OS and continued until death, end of follow-up (31 December 2015) or until a diagnosis of a SPN was made, whichever came first. If an individual had two SPNs, only the first SPN was included in the analysis of overall risk. However, for the analysis of specific SPNs, subsequent primary neoplasms beyond the second neoplasm were also assessed. Four individuals had the same morphology code for the primary and subsequent neoplasms. Three of these individuals were diagnosed with a primary OS and the subsequent malignancies, all of which occurred within 24 months, were also osteosarcomas. There was also one ES patient with a subsequent tumour classified as a new ES. Synchronous (at time of diagnosis) or metachronous (separated in time) OS, occasionally seen among patients with a predisposing syndrome, are rare manifestations of OS, where two or more skeletal lesions are seen without the presence of metastasis in the lungs or other non-skeletal foci.^15^ It is controversial whether multiple skeletal OS are two distinct clonally unrelated tumours or if they represent one primary tumour with skeletal metastasis.^16^ For this reason, the SPNs in these four cases were regarded as metastatic disease rather than new primaries.

### Statistical analysis

Data regarding age- (five-year classes), sex- and calendar year-specific incidence rates relative to the Swedish population for overall and specific cancer types were retrieved from the National Board of Health and Welfare.^17^ The number of expected cancer cases was calculated by multiplying the Swedish incidence rates by the total person-time at risk for each corresponding age, sex, and calendar year stratum in our cohort. Overall and cancer type-specific standardised incidence ratios (SIRs) were estimated by dividing the observed number of cancer cases by the number of expected cases. SIRs were calculated stratified by sex, age at diagnosis, years since ES or OS diagnosis, calendar year at ES or OS diagnosis and site of primary tumour. The absolute excess risk (AER) was defined as the observed minus expected number of cancers divided by person-years at risk multiplied by 10,000. Exact 95% confidence intervals for SIR and AER estimates were calculated assuming that the number of observed cases followed a Poisson distribution. In analyses limited to our cohort we estimated the cumulative incidence of overall cancer stratified by ES or OS diagnosis. Here, death was considered as a competing event.

Patients with a malignancy prior to ES or OS diagnosis were believed to represent a high-risk group for SPN. A sensitivity analysis was therefore conducted by excluding patients with a previous history of cancer prior to their primary bone tumour malignancy (Supplementary Table A).

## Results

### Characteristics of the cohort

Through December 2015, 115 SPNs were diagnosed in 104 out of the 1779 patients in the cohort. Fourteen patients had two SPNs and two patients had three SPNs. The total follow-up time was 16,170 person-years (median 2.6 years; range 0–58 years). The most commonly observed SPNs were breast cancer (*n* = 18) followed by genitourinary cancer (*n* = 17), skin cancer (melanoma or squamous cell carcinoma) (*n* = 13), CNS neoplasm (*n* = 11), sarcoma (other than the primary bone sarcoma type) (*n* = 10) and female genital malignancy (*n* = 10).

Eighty-six patients (5%) had suffered a malignancy prior to their primary diagnosis of OS or ES: seventy-nine (7%) of the OS patients and 7 (1%) of the ES patients (Table 1). The most commonly occurring malignancies prior to the primary bone sarcoma diagnosis were breast cancer (*n* = 18), skin cancer (*n* = 18; nine melanomas and nine squamous cell carcinomas), genitourinary malignancies (*n* = 16) and female genital malignancies (*n* = 15). Two patients were diagnosed with retinoblastoma and three patients with a sarcoma (two soft tissue sarcomas and one chondrosarcoma) prior to the primary bone sarcoma diagnosis. The median time from the previous malignancy to the primary bone sarcoma diagnosis was 6 years (range 0–42 years).

**Table 1:** Characteristics of the osteosarcoma and ewing sarcoma cohorts.

		Osteosarcoma		Ewing sarcoma		Total	
No. of patients		1201		578		1779	
Sex							
Male		705 (59%)		358 (62%)		1063 (60%)	
Female		496 (41%)		220 (38%)		716 (40%)	
Age at diagnosis^a^		21.8 (15.3–54.9)		17.4 (12–23.2)		19.4 (14.3–40.9)	
0–9 years		87 (7%)		104 (18%)		188 (11%)	
10–19 years		471 (39%)		273 (47%)		744 (42%)	
≥20 years		646 (54%)		201 (35%)		847 (48%)	
Calendar year at diagnosis							
1958–1979		474 (40%)		167 (29%)		641 (36%)	
1980–1999		399 (33%)		214 (37%)		613 (35%)	
2000–2015		328 (27%)		197 (34%)		525 (30%)	
Site							
Extremity		979 (82%)		304 (53%)		1283 (72%)	
Unspecified		41 (3%)		24 (4%)		65 (4%)	
Central		181 (15%)		250 (43%)		431 (24%)	
Spine^b^		26 (14%)		31 (12%)		57 (13%)	
Pelvic^b^		104 (58%)		136 (54%)		24 (56%)	
Tissue							
Bone		1135 (95%)		495 (86%)		1630 (92%)	
Soft tissue		66 (5%)		83 (14%)		149 (8%)	
Malignancy prior to primary bone cancer		79 (7%)		7 (1%)		86 (5%)	
Follow-up (years)^a^		2.5 (0–57.8)		2.9 (0–56.3)		2.6 (0–57.8)	
Time to SPN (years)^a^		17.1 (0–52.8)		17.0 (0−48.7)		17.1 (0–52.8)	

^a^Median, range in parenthesis.

^b^Percentage of centrally located tumours.

### Risk of subsequent primary neoplasms

**Fig. 1:**
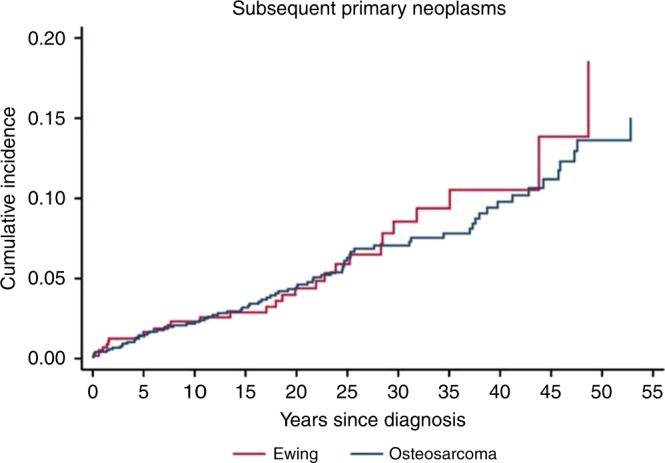
Cumulative incidence of subsequent primary neoplasms after initial bone sarcoma diagnosis.

ES survivors were 4.2 (95% CI, 2.8–6.1) and OS survivors 1.9 (95% CI, 1.5–2.4) times more likely to develop a SPN than expected. By age at primary tumour diagnosis, the highest SIR among ES survivors was evident in the age group 10–19 years at diagnosis, with a 7.2-fold increased risk for a SPN compared to the general population (95% CI, 4.2–11.6). For OS survivors, the highest SIR was in patients aged 0–9 years at diagnosis who were 6.7 times more likely to develop a SPN than expected (95% CI, 1.8–17.2). By anatomic site, the highest SIR was observed for pelvic ES (SIR 7.6; 95% CI, 2.1–19.4). The risk was not lower for patients treated in later eras (SIR_2000-2015_ 2.0; 95% CI, 1.1–3.5) compared with earlier treatment eras (SIR_1958-1979_ 2.0; 95% CI, 1.5–2.6) (Table 2). Over 30 years of follow-up, the cumulative incidences of SPNs were 7.1% and 8.6% for OS and ES patients, respectively (Fig. 1). The median time from primary tumour diagnosis to SPN was 17 years. The overall SIR for any SPN was 2.2 (95% CI, 1.8–2.7) and 2.1 (95% CI, 1.7–2.6) when survivors with a prior history of cancer were included respectively excluded from the analysis (Supplementary Table A). The overall SIR for SPNs among the 86 survivors with a previous history of a cancer was 3.0 (95% CI, 1.4–5.7).

**Table 2:** Standardised Incidence Ratios (SIRs) and Absolute Excess Risks (AERs) of subsequent primary neoplasms among patients with bone sarcoma in Sweden by calendar year at diagnosis, age at diagnosis, site and follow-up.

	All				Osteosarcoma			Ewing sarcoma		
	Person years	No. Obs/exp	SIR (95% CI)	AER (95% CI)	No. Obs/exp	SIR (95% CI)	AER (95% CI)	No. Obs/exp	SIR (95% CI)	AER (95% CI)
Overall	16,170	104/47	2.2 (1.8–2.7)	35.1 (23.3–48.7)	75/40.4	1.9 (1.5–2.3)	30.7 (16.5–47.5)	29/6.8	4.2 (2.8–6.1)	45.4 (25.8–71.3)
Sex										
Male	9332	48/25.6	1.9 (1.4–2.5)	24.0 (10.5–40.1)	38/22.4	1.7 (1.2–2.3)	24.5 (7.1–46.7)	10/3.3	3.1 (1.5–5.7)	22.8 (5.2–51.2)
Female	6839	56/21.6	2.6 (2.0–3.4)	50.3 (30.2–74.7)	37/18.0	2.1 (1.4–2.8)	38.6 (16.3–67.1)	19/3.6	5.3 (3.2–8.3)	80.0 (40.7–135)
Calendar year at diagnosis										
1958–1979	7003	50/25.3	2.0 (1.5–2.6)	35.3 (16.9–58.0)	36/22.4	1.6 (1.1–2.2)	25.1 (5.2–50.5)	14/2.9	4.8 (2.6–8.1)	70.9 (30.4–132)
1980–1999	6554	41/15.6	2.6 (1.9–3.6)	38.8 (21.1–61.1)	29/12.7	2.2 (1.5–3.3)	38.4 (15.8–68.2)	12/2.9	4.1 (2.1–7.2)	39.5 (14.3–78.3)
2000–2015	2613	13/6.4	2.0 (1.1–3.5)	25.4 (2.1–60.7)	10/5.3	1.9 (0.9–3.5)	29.2 (−3.3 to 81.6)	3/1.1	2.9 (0.6–8.3)	19.3 (-4.3 to 76.3)
Age at diagnosis										
0–9 years	2125	8/1.2	6.5 (2.8–12.8)	31.9 (10.5–68.4)	4/0.6	6.7 (1.8–17.2)	34.2 (5.0–96.9)	4/0.6	6.3 (1.7–16.2)	29.8 (4.0–85.0)
10–19 years	8020	37/10.4	3.6 (2.5–4.9)	33.2 (19.5–50.6)	20/8.0	2.5 (1.5–3.8)	21.0 (7.3–40.2)	17/2.4	7.2 (4.2–11.6)	62.8 (32.4–107)
≥20 years	6025	59/35.6	1.7 (1.3–2.1)	38.8 (15.5–67.2)	51/31.7	1.6 (1.2–2.1)	41.8 (13.5–76.7)	8/3.9	2.1 (0.9–4.1)	29.2 (−2.8 to 83.8)
Site										
Extremity	12,683	80/38.6	2.1 (1.6–2.6)	32.7 (19.6–48.1)	63/34.5	1.8 (1.4–2.3)	28.5 (13.9–46.1)	17/4.1	4.2 (2.4–6.7)	48.1 (21.7–86.1)
Central	2949	19/6.7	2.9 (1.7–4.5)	41.9 (16.2–78.1)	9/4.4	2.0 (0.9–3.9)	48.3 (−3.2 to 134)	10/2.2	4.5 (2.2–8.3)	38.8 (12.8–80.7)
Pelvic	1119	7/1.9	3.8 (1.5–7.8)	45.9 (8.5–112)	3/1.3	2.3 (0.5–6.6)	49.5 (−21.1 to 221)	4/0.5	7.6 (2.1–19.4)	44.4 (7.2–124)
Non-pelvic central	1830	12/4.8	2.5 (1.3–4.4)	39.4 (7.7–88.4)	6/3.1	1.9 (0.7–4.2)	47.7 (−14.6 to 163)	6/1.7	3.5 (1.3–7.7)	35.2 (4.1–93.1)
Follow-up, years										
0–5	5085	25/10.5	2.4 (1.5–3.5)	28.5 (11.2–51.9)	16/9.4	1.7 (1.0–2.8)	19.7 (−0.8 to 49.6)	9/1.1	8.4 (3.8–15.9)	45.4 (17.4–91.7)
5–30	9292	58/22.3	2.6 (2.0–3.4)	38.4 (23.4–56.7)	42/18.7	2.2 (1.6–3.0)	35.8 (17.8–58.4)	16/3.6	4.4 (2.5–7.2)	44.7 (20.0–80.7)
>30	1793	21/14.5	1.5 (0.9–2.2)	36.5 (−8.1 to 98.4)	17/12.3	1.4 (0.8–2.2)	32.9 (−16.8 to 105)	4/2.2	1.9 (0.5–4.8)	50.4 (−28.9 to 221)

*SIR* standardised incidence ratio, *AER* absolute excess risk (mean excess subsequent primary neoplasms per 10,000 person-years), *CI* confidence interval.

With regard to specific SPNs, the overall SIR for subsequent female genital malignancies was 3.3 times more than expected (SIR 3.3; 95% CI, 1.6–6.0) with an AER of 9.7 cases per 10,000 person-years (95% CI, 2.4–21.5). Only OS survivors demonstrated increased risks for CNS neoplasms (SIR 6.4; 95% CI, 3.2–11.4) and skin cancer (2.5; 95% CI, 95% CI, 1.3–4.3). The overall SIR (SIR 0.8; 95% CI, 0.3–1.6) for digestive tract malignancies was not higher than expected (Table 3). As for genitourinary malignancies, only when the primary bone tumour was located in the pelvis was the observed number of subsequent genitourinary malignancies significantly higher than expected (SIR 6.6; 95% CI, 1.4–19.4) with an AER of 22.3 cases per 10,000 person-years (95% CI, 1.5–72.7) (Supplementary Table B).

**Table 3:** Standardised incidence ratios and absolute excess risks of subsequent primary neoplasm subtypes among patients with bone sarcoma in Sweden.

	Overall				Osteosarcoma			Ewing sarcoma		
Types of subsequent primary neoplasms	Person years	No. Obs/exp	SIR (95% CI)	AER (95% CI)	No. Obs/exp	SIR (95% CI)	AER (95% CI)	No. Obs/exp	SIR (95% CI)	AER (95% CI)
Any	19,170	104/47.2	2.2 (1.8–2.7)	35.1 (23.3–48.7)	75/40.4	1.9 (1.4–2.5)	30.7 (16.5–47.5)	29/6.8	4.2 (2.8–6.1)	45.4 (25.8–71.3)
Breast	7169	18/7.1	2.5 (1.5–4.0)	15.2 (5.0–29.8)	12/5.8	2.1 (1.1–3.6)	12.1 (0.7–29.6)	6/1.3	4.7 (1.7–10.2)	23.0 (4.5–57.3)
Bone	16,905	2/0.2	13.7 (1.7–49.7)	1.1 (0.1–4.2)	0/0.1	–	–	2/0.0	47.7 (5.8–172)	3.9 (0.4-14.2)
Soft tissue	16,826	8/0.4	20.6 (8.9–40.5)	4.5 (1.8–9.1)	3/0.3	9.5 (2.0–27.8)	2.3 (0.3–7.2)	5/0.1	67.3 (21.9–157)	9.8 (3.1–23.1)
Haematologic	16,874	10/4.4	2.3 (1.1–4.2)	3.3 (0.2–8.3)	6/3.7	1.6 (0.6–3.5)	1.9 (−1.3 to 7.9)	4/0.7	5.6 (1.5–14.4)	6.5 (0.7–18.9)
Skin	16,793	13/5.7	2.3 (1.2–3.9)	4.3 (0.7–9.8)	12/4.9	2.5 (1.3–4.3)	6.1 (1.1–13.7)	1/0.9	1.2 (0.0–6.5)	0.3 (−1.6 to 9.3)
CNS	16,815	11/2.3	4.8 (2.4–8.6)	5.2 (1.9–10.3)	11/1.8	6.3 (3.1–11.2)	7.9 (3.2–15.3)	0/0.5	–	–
Digestive tract	16,877	7/9.4	0.7 (0.3–1.5)	−1.4 (−3.9 to 3.0)	5/8.4	0.6 (0.2–1.4)	−2.9 (−5.7 to 2.8)	2/1.0	2.0 (0.2–7.2)	2.0 (−1.5 to 12.3)
Genitourinary	16,838	17/11.8	1.4 (0.8–2.3)	3.1 (−1.1 to 9.2)	15/10.5	1.4 (0.8–2.4)	3.8 (−1.8 to 12.1)	2/1.3	1.5 (0.2–5.6)	1.4 (−2.1 to 11.7)
Female genital	7128	10/3.2	3.1 (1.5–5.8)	9.5 (2.4–21.5)	7/2.6	2.7 (1.1–5.5)	8.6 (0.3–23.2)	3/0.6	5.4 (1.1–15.7)	11.9 (0.3–40.0)
Other	16,846	19/7.6	2.5 (1.5–3.9)	6.8 (2.3–13.1)	13/6.6	2.0 (1.1–3.4)	5.5 (0.3–13.3)	6/1.0	6.0 (2.2–13.1)	9.9 (2.4–23.9)

*SIR* standardised incidence ratio, *AER* absolute excess risk (mean excess subsequent primary neoplasms per 10,000 person-years), *CI* confidence interval.

### Breast cancer

With respect to primary tumour type, ES survivors were 4.7 and OS survivors 2.1 times more likely than the general population to develop a subsequent breast cancer (SIR_ES_ 4.7; 95% CI, 1.7–10.2 and SIR_OS_ 2.1; 95% CI 1.1–3.6) (Table 4). The risk remained increased after 30 years of follow-up and for ES survivors with more than 30 years of follow-up an excess of 127 breast cancers per 10,000 years was estimated. Regarding anatomical site the overall SIR for a subsequent breast cancer was more than three times as high for a primary bone sarcoma with a central location compared with a location in the extremity (SIR_central_ 6.5; 95% CI, 2.1–15.2 and SIR_extremity_ 2.1; 95% CI, 1.1–3.6). The highest SIR was evident for ES survivors with a centrally but non-pelvic primary ES location (SIR, 11.6; 95% CI, 2.4–34.0). No breast cancers were recorded among pelvic ES survivors. Due to small numbers, any trend in risk over calendar period was difficult to evaluate. The median time from primary diagnosis to the development of a breast cancer was 27 (1–49) years for ES- and 23 (3–46) years for OS survivors.

**Table 4:** Standardised incidence ratios and absolute excess risks of subsequent breast cancer among patients with bone sarcoma by calendar year at diagnosis, age at diagnosis, site and follow-up.

	All			Osteosarcoma			Ewing sarcoma		
	No. Obs/exp	SIR (95% CI)	AER (95% CI)	No. Obs/exp	SIR (95% CI)	AER (95% CI)	No. Obs/exp	SIR (95% CI)	AER (95% CI)
Calendar Year									
1958–1979	11/4.0	2.7 (1.4–4.9)	21.6 (4.6–48.4)	8/3.3	2.4 (1.1–4.8)	19.7 (0.7–52.1)	3/0.7	4.2 (0.9–12.3)	27.0 (−1.1 to 95.2)
1980–1999	6/2.4	2.5 (0.9–5.5)	13.2 (−0.6 to 38.8)	3/1.9	1.6 (0.3–4.6)	5.6 (−6.6 too 35.2)	3/0.5	6.5 (1.3–19.1)	31.4 (2.0–103)
2000–2015	1/0.8	1.3 (0.0–7.5)	2.2 (−6.2 to 41.3)	1/0.6	1.6 (0.0–8.7)	4.7 (−8.0 to 64.1)	0/0.1	-	-
Age at diagnosis									
0–9 years	1/0.2	6.7 (0.2–37.5)	9.6 (−1.4 to 61.2)	0/0.1	-	-	1/0.1	11.9 (0.3–66.3)	17.4 (−1.1 to 104)
10–19 years	7/1.8	3.9 (1.5–7.9)	15.2 (2.9–36.9)	4/1.4	2.8 (0.8–7.2)	10.4 (−1.3 to 35.4)	3/0.4	7.4 (1.5–21.8)	28.1 (2.3–90.5)
≥20 years	10/5.1	1.9 (0.9–3.6)	17.0 (−1.2 to 46.2)	8/4.4	1.8 (0.8–3.6)	16.2 (−4.0 to 50.5)	2/0.8	2.5 (0.3–9.1)	20.0 (−9.1 to 106)
Site									
Extremity	13/6.2	2.1 (1.1–3.6)	11.4 (1.2–26.8)	10/5.3	1.9 (0.9–3.5)	10.2 (−1.0 to 28.2)	3/0.9	3.2 (0.7–9.3)	15.5 (−2.5 to 59.0)
Central	5/0.8	6.5 (2.1–15.2)	40.5 (8.2–104)	2/0.4	4.6 (0.6–16.5)	48.3 (−6.0 to 210)	3/0.3	9.0 (1.9–26.4)	36.9 (4.0–117)
Pelvic	1/0.2	4.6 (0.1–25.4)	20.8 (−5.2 to 143)	1/0.2	6.9 (0.2–38.3)	67.7 (−9.5 to 430)	0/0.1	–	–
Non-pelvic central	4/0.6	7.3 (2.0–18.6)	51.4 (8.1–144)	1/0.3	3.4 (0.1–19.1)	35.9 (−13.5 to 268)	3/0.3	11.6 (2.4–34.0)	57.9 (7.6–180)
Follow-up, years									
0–5	3/1.2	2.6 (0.5–7.6)	8.7 (−2.5 to 35.8)	2/1.0	2.0 (0.2–7.1)	6.8 (−5.3 to 42.9)	1/0.1	7.3 (0.2–40.7)	12.7 (−1.6 to 80.3)
5–30	9/3.7	2.5 (1.1–4.7)	12.6 (1.1–31.7)	7/3.0	2.3 (0.9–4.8)	13.1 (−0.7 to 37.4)	2/0.7	3.1 (0.4–11.0)	11.4 (−3.5 to 55.7)
>30	6/2.3	2.6 (1.0–5.7)	45.5 (−1.0 to 132)	3/1.8	1.7 (0.3–4.9)	19.5 (−19.1 to 113)	3/0.5	6.1 (1.3–17.9)	127 (6.6–419)

*SIR* standardised incidence ratio, *AER* absolute excess risk (mean excess subsequent primary neoplasms per 10,000 person-years), *CI* confidence interval.

### Secondary sarcoma (bone and soft tissue)

The overall risk for developing a secondary soft tissue sarcoma was nearly 21-fold that of the general population (SIR 20.6; 95% CI, 8.9–40.5) (Table 3). The risk was highest among ES survivors who were seven times more likely than OS patients and 67 times more likely than the general population to develop a soft tissue sarcoma (SIR_ES_ 67.3; 95% CI, 21.9–157 and SIR_OS_ 9.5; 95% CI, 2.0–27.9). Eight subsequent soft tissue sarcomas developed after a median of 18 and 19 years for ES and OS survivors, respectively. Two secondary osteosarcomas were observed in 2 ES survivors and no secondary bone sarcomas were seen among the OS patients. The median time to development of a subsequent bone sarcoma was 8 years.

### Haematological malignancies

The rate of a subsequent haematological malignancy was 5.6 (95% CI, 1.5–14.4) and 1.6 (95% CI, 0.6–3.5) times that expected among ES and OS survivors respectively (Table 3). The SIR was highest for primary bone sarcoma patients in the age group 0-9 years (SIR 11.0; 95% CI, 1.3–39.7). By follow-up, the highest overall SIR was within 5 years after diagnosis (SIR 6.5; 95% CI, 2.4–14.5) (Supplementary Table C). The subsequent haematological malignancies included seven leukaemias (four acute myeloid leukaemias, one chronic myeloid leukaemia, one acute lymphatic leukaemia and one chronic lymphatic leukaemia), two lymphomas and one myeloma. The median time from primary diagnosis to subsequent haematologic malignancy was 3 and 14 years for ES and OS survivors, respectively.

## Discussion

This large-scale population-based study is the first of its kind to demonstrate that even after 30 years of follow-up, ES survivors are at a significantly higher risk than the general population for developing a subsequent breast cancer. Due to the high incidence of breast cancer in the general population the excess risk after 30 years of follow-up is especially noteworthy. This result stands in contrast to the British Childhood Cancer Survivor Study (BCCSS), which did not demonstrate an increased risk for a subsequent breast or any other SPN among ES and OS survivors after 30 years of follow-up, perhaps due to smaller sample size. Significantly elevated risks for subsequent breast cancers in the present study were most apparent for patients treated at age 10–19 years and for centrally located tumours. Although not consistent in different paediatric cancer cohorts these data support the notion that females treated at pubertal age, when breast tissue proliferates, have the highest risk for a subsequent breast cancer.^2,9,18–20^ The excess cancer risk evident in the present study was largely driven by breast cancer, but the significant contribution to the excess risk by female genital malignancies is a novel finding. This observation may indicate a possible role for BRCAness among bone sarcoma survivors acquiring SPNs.^21^ Somatic BRCA-like traits driving tumorigenesis has been shown in a study on OS tumour samples by Kovacs et al.^22^ However, no correlation between bone sarcoma and female genital malignancies was found in the SEER (Surveillance Epidemiology End-Result) population-based cancer registry in the US, but this study had a smaller sample size and fewer SPNs than in the current study.^4^

Another important finding in this study is that the enhanced risk for SPNs remains elevated for patients treated during recent years, a finding that was also noted in the CCSS cohort.^6^ In the latter study, there was a reduction in SPNs among childhood cancer survivors treated in the 1990s compared with those diagnosed in the 1970s; a reduction that correlated clearly with a reduction in radiation dose.^6^ However, when stratified into individual malignancies, the risk was only significantly reduced for Hodgkin lymphoma. The use of RT was reduced from 77% to 34% of patients during the study period. More restricted use of RT was also noted in a Children’s Oncology Group study, including ES patients treated from 1996-98 in which 65% of the patients were treated without RT.^23^ The reason that the risk for SPNs remains high for the ES survivors in the current study is unknown. An explanation could be that the use of RT has not changed substantially over the time period studied and that RT is still a significant cause of SPNs. Lastly, there may also be other factors that drive the high risk of SPNs among ES and OS survivors.

The ES- and OS-specific SIRs and AERs for patients younger than 20 years in the present study correspond well to the results reported by the North American CCSS and the aforementioned BCCSS.^2,6,24^ The BCCSS is a population-based national childhood cancer registry study encompassing 664 individuals diagnosed with bone cancer between 1940 and 1991.^24^ Although not population-based but unique due to the size, long follow-up and detailed treatment information, the CCSS study included 1205 OS and 714 ES patients diagnosed before the age of 21 years.^2,6^ Another population-based Swedish study performed more than 10 years ago estimated SIRs of 1.5 and 5.6 for OS and ES survivors, respectively.^25^ The SEER study (overlapping cohorts with the CCSS) reported SIRs of 12.9 and 5.6 for ES and OS survivors, respectively, although the AERs were lower than in the present study.^4^ Studies of cumulative incidence of SPNs in ES cohorts have reported great variations, with the risk varying from 0.9% at five years to 42.8% at 20 years, but the competing risk of death has not always been accounted for.^5,26–33^ The cumulative incidences of SPN at 30 years, available in the aforementioned CCSS and BCCSS cohorts,^2,6,24^ were strikingly similar to the results in the present study.

The increased risk for SPNs among OS and ES survivors is likely to have different aetiologies. For OS survivors the excess risk can be partly attributed to a genetic predisposition. Li Fraumeni, hereditary retinoblastoma and Rothmund-Thompson syndrome are associated with OS and other malignancies including breast and skin cancer.^3,34–37^ To our knowledge there is no such genetic predisposition associated with ES. Moreover, nearly 7% of the OS patients in this cohort were diagnosed with another malignancy prior to OS diagnosis, whereas this was only the case for 1.2% of the ES patients. This indicates that a number of OS patients in our cohort harbour a cancer predisposition syndrome, or have received treatment prior to primary OS diagnosis with alkylating agents, anthracyclines or RT, all of which may cause OS.^38,39^ Apart from genetic predisposition in OS patients, differences in the use of RT may be a major reason for the differences observed in SPNs in this study. Radiotherapy has traditionally been considered mainstay of local treatment of ES. OS on the contrary, is regarded as a RT- resistant malignancy, and therefore RT has been restricted to non-resectable or incompletely resected tumours associated with poor prognosis,  where few patients will survive long enough to acquire a SPN. The use of RT in the treatment of primary ES is debated and has led to different local treatment strategies in the US and Europe.

This study is limited by the lack of treatment information. The Swedish Cancer Registry does not encompass information about treatment and there is no clinical registry available for linkage that spans as far back as the beginning of this study period. One of the strengths of this study includes the solid quality of the Registry and the length of the study period. The quality of the data relies on the unique personal identification number available for all Swedish citizens, and the multiple sources for reporting to the register. Adding treatment variables such as type and dose of chemotherapy and type and site of RT would be very useful but would likely be at the expense of the quality of the Registry. Assessing individual patient records would be a possibility, but due to the long study period it is likely that detailed treatment information still would be lacking to a large extent. Other studies on ES survivors with information on treatment have still had difficulties comparing patients treated with and without RT since the majority (75–90%) of ES survivors have been treated with RT, thus rendering the non-RT group too small for meaningful analysis.^24,40^ Another concern associated with the length of the study period is the changes in treatment that have occurred over time. However, the risk was not lower for patients treated later during the study period, after which conformal radiation techniques such as intensity modulated- and image guided techniques were introduced. Regarding chemotherapy, the regimes have been unchanged for the past 40 years.

Some of the excess risk observed in this study may be due to surveillance bias. Basal cell carcinomas and non-glioma CNS tumours are known to be underestimated in the general population due to their indolent course.^3^ However, basal cell carcinomas are not registered in the Swedish Cancer Registry and all SPNs except for 3 meningiomas observed in this study were invasive malignancies unlikely to be missed. Furthermore, brain imaging is not part of routine follow-up of OS patients.

As the number of childhood cancer survivors increases, so does the need for guidelines for long-term follow-up of treatment related side-effects. Two out of three survivors will develop at least one late onset therapy related complication.^41^ The Children’s Oncology Group in the United States have developed comprehensive exposure related guidelines (COG-LTFU guidelines) aiming at early detection and timely intervention of late effects such as SPNs.^42^ However, as stated by the ESMO-PaedCan-EUROCAN guidelines, there is a lack of consensus among experts on optimal follow-up recommendations.^43^ We believe the current study provides important data which will improve current guidelines for surveillance among patients who have survived primary bone sarcoma.

In conclusion, this study shows that ES and OS survivors are at increased risk for developing specific SPNs, a risk that remains elevated with continued long-term follow-up, especially for female ES patients who have substantial risk for subsequent breast cancer even after 30 years. Early and continued screening for breast cancer among females is motivated as ES and OS survivors reach the age at which the breast cancer incidence becomes significant in the general population. Furthermore, an association between primary bone sarcoma and subsequent female genital malignancies was shown in this study. The significant contribution of breast cancer and female genital malignancies to the excess cancer risk suggests a possible role of BRCA-associated genetic aberrations among female bone sarcoma patients. Lastly, this study demonstrates that the increased cancer risk that bone sarcoma patients face, remains high even with modern treatment regimens.

## Supplementary information

Supplementary tables

## Data Availability

The data that support the findings of this study are available from the Cancer Registry at Socialstyrelsen (https://www.socialstyrelsen.se/en/search-results/?q=cancer+registry). Restrictions apply to the availability of these data, which were used under license for this study. Data are available from the authors with permission from Socialstyrelsen (National Board of Health and Welfare).
