# Supplementary material for: Subsequent primary neoplasms among bone sarcoma survivors; increased risks remain after 30 years of follow-up and in the latest treatment era, a nationwide population-based study
Source: Br J Cancer. 2020 Feb 18;122(8):1242–9. doi: 10.1038/s41416-020-0748-3 (PMC7156510; doi:10.1038/s41416-020-0748-3)
Supplement: Supplementary file 1 — Supplementary tables [file 41416_2020_748_MOESM1_ESM.docx]

| **Table A. Standardized Incidence Ratios of subsequent primary neoplasm subtypes among patients with Bone Sarcoma in Sweden excluding prior neoplasms** | | | | | | | | | | | | |
| --- | --- | --- | --- | --- | --- | --- | --- | --- | --- | --- | --- | --- |
|  | **Overall** | | **Overall**  **Excluding prior neoplasms** | | **Osteosarcoma** | | **Osteosarcoma**  **Excluding prior neoplasms** | | **Ewing sarcoma** | | **Ewing sarcoma**  **Excluding prior neoplasms** | |
| **Types of subsequent primary neoplasms** | **No. Obs/exp** | **SIR**  **(95% CI)** | **No. Obs/exp** | **SIR**  **(95% CI)** | **No. Obs/exp** | **SIR**  **(95% CI)** | **No. Obs/exp** | **SIR**  **(95% CI)** | **No. Obs/exp** | **SIR**  **(95% CI)** | **No. Obs/exp** | **SIR**  **(95% CI)** |
| **Any** | 104/47.2 | 2.2  (1.8–2.7) | 95/44.2 | **2.1**  **(1.7–2.6)** | 75/40.4 | **1.9**  **(1.5–2.3)** | **67/37.4** | **1.8**  **(1.4–2.3)** | **29/6.8** | **4.2**  **(2.8–6.1)** | **28/6.8** | **4.1**  **(2.8–6.0)** |
| **Breast** | 18/7.1 | 2.5  (1.5–4.0) | 17/6.6 | **2.6**  **(1.5–4.1)** | 12/5.8 | **2.1**  **(1.1–3.6)** | **11/5.3** | **2.1**  **(1.0–3.7)** | **6/1.3** | **4.7**  **(1.7–10.2)** | **6/1.2** | **4.8**  **(1.8–10.6)** |
| **Bone** | **2/0.2** | **13.7**  **(1.7–49.7)** | **2/0.1** | **14.1**  **(1.7–50.8)** | 0/0.1 | **–** | **0/0.1** | **–** | **2/0.0** | **47.7**  **(5.8–172)** | **2/0.0** | **47.9**  **(5.8–173)** |
| **Soft tissue** | **8/0.4** | **20.6**  **(8.9–40.5)** | **7/0.4** | **19.1**  **(7.7–39.3)** | 3/0.3 | **9.5**  **(2.0–27.8)** | **2/0.3** | **6.8**  **(0.8–24.6)** | **5/0.1** | **67.3**  **(21.9–157)** | **5/0.1** | **68.1**  **(22.1–159** |
| **Haematologic** | **10/4.4** | **2.3**  **(1.1–4.2)** | **7/4.1** | **1.7**  **(0.7–3.5)** | **6/3.7** | **1.6**  **(0.6–3.5)** | **4/3.4** | **1.2**  **(0.3–3.0)** | **4/0.7** | **5.6**  **(1.5–14.4)** | **3/0.7** | **4.2**  **(0.9–12.4)** |
| **Skin** | **13/5.7** | **2.3**  **(1.2–3.9)** | **11/5.3** | **2.1**  **(1.0–3.7)** | **12/4.9** | **2.5**  **(1.3–4.3)** | **10/4.4** | **2.3**  **(1.1–4.2)** | **1/0.9** | **1.2**  **(0.0–6.5)** | **1/0.9** | **1.2**  **(0.0–6.5)** |
| **CNS** | **11/2.3** | **4.8**  **(2.4–8.6)** | **10/2.2** | **4.6**  **(2.2–8.4)** | **11/1.8** | **6.3**  **(3.1–11.2)** | **10/1.7** | **6.0**  **(2.9–11.0)** | **0/0.5** | **–** | **0/0.5** | **–** |
| **Digestive tract** | **7/9.4** | **0.7**  **(0.3–1.5)** | **6/8.5** | **0.7**  **(0.3–1.5)** | 5/8.4 | **0.6**  **(0.2–1.4)** | **4/7.5** | **0.5**  **(0.1–1.4)** | **2/1.0** | **2.0**  **(0.2–7.2)** | **2/1.0** | **2.0**  **(0.2–7.4)** |
| **Genitourinary** | **17/11.8** | **1.4**  **(0.8–2.3)** | **15/10.8** | **1.4**  **(0.8–2.3)** | 15/10.5 | **1.4**  **(0.8–2.4)** | **14/9.5** | **1.5**  **(0.8–2.5)** | **2/1.3** | **1.5**  **(0.2–5.6)** | **1/1.3** | **0.8**  **(0.0–4.3)** |
| **Female genital** | **10/3.2** | **3.1**  **(1.5–5.8)** | **10/2.9** | **3.4**  **(1.7–6.3)** | **7/2.6** | **2.7**  **(1.1–5.5)** | 7/2.4 | **2.9**  **(1.2–6.1)** | **3/0.6** | **5.4**  **(1.1–15.7)** | **3/0.5** | **5.7**  **(1.2–16.7)** |
| **Other** | **19/7.6** | **2.5**  **(1.5–3.9)** | **18/6.9** | **2.6**  **(1.5–4.1)** | **13/6.6** | **2.0**  **(1.1–3.4)** | 12/6.0 | **2.0**  **(1.0–3.5)** | **6/1.0** | **6.0**  **(2.2–13.1)** | **6/0.1** | **6.1**  **(2.2–13.3)** |
| **Table A (Continued). Standardized Incidence Ratios and Absolute Excess Risks of subsequent primary neoplasm subtypes among patients with Bone Sarcoma in Sweden excluding prior neoplasms** | | | | | | | | | | | | |
| **Abbreviations:**  **SIR=standardized incidence ratio**  **CI=confidence interval** | | | | | | | | | | | | |

| **Table B. Standardized Incidence Ratios and Absolute Excess Risks for Soft Tissue, Genitourinary and Female Genital Subsequent Neoplasms by Calendar Year, Age at Diagnosis, Site and Follow-up** | | | | | | | | | |
| --- | --- | --- | --- | --- | --- | --- | --- | --- | --- |
|  | **Soft tissue** | | | **Genitourinary** | | | **Female Genital** | | |
|  | **No. obs/exp** | **SIR (95% CI)** | **AER (95% CI)** | **No. Obs/exp** | **SIR (95% CI)** | **AER (95% CI)** | **No. obs/exp** | **SIR (95% CI)** | **AER (95% CI)** |
| **Calendar Year**  **1958-1979** | 6/0.2 | 31.4  (11.5–68.4) | 7.9  (2.7–17.5) | 6/6.2 | 1.0  (0.4–2.1) | -0.3  (-5.4–9.3) | 4/1.8 | 2.2  (0.6–5.6) | 6.7  (-2.3–25.9) |
| **1980-1999** | 2/0.2 | 13.5  (1.6–48.7) | 2.7  (0.1–10.3) | 8/3.9 | 2.1  (0.9–4.0) | 6.0  (-0.7–17.4) | 6/1.1 | 5.7  (2.1–12.3) | 18.2  (4.2–44.3) |
| **2000-2015** | **0/0.1** | **–** | **–** | **3/1.7** | **1.8**  **(0.4–5.3)** | **5.1**  **(-4.0–27.0)** | **0/0.3** | **–** | **–** |
| **Age at Diagnosis**  **0–9 years** | **0/0.0** | **–** | **–** | **0/0.2** | **–** | **–** | **1/0.1** | **16.2**  **(0.4–90.0)** | **10.6**  **(-0.4–62.4)** |
| **10-19 years** | **7/0.1** | **58.4**  **(23.5–120)** | **8.3**  **(3.3–17.3)** | **2/5.5** | **0.8**  **(0.1–2.9)** | **-0.6**  **(-2.7–5.7)** | **7/0.7** | **10.7**  **(4.3–22.0)** | **19.0**  **(6.5–41.3)** |
| **≥ 20 years** | **1/0.3** | **4.0**  **(0.1–22.5)** | **1.2**  **(-0.3–8.3)** | **15/9.1** | **1.7**  **(0.9–2.7)** | **9.4**  **(-1.1–24.7)** | **2/2.5** | **0.8**  **(0.1–2.9)** | **-1.7**  **(-7.7–16.3)** |
| **Site**  **Extremity** | **8/0.1** | **25.8**  **(11.1–50.8)** | **5.8**  **(2.4–11.8)** | **12/9** | **1.3**  **(0.7–2.3)** | **2.3**  **(-2.1–9.1)** | **7/2.8** | **2.5**  **(1.0–5.1)** | **7.0**  **(0.0–19.4)** |
| **Central** | **0/0.1** | **–** | **–** | **4/2** | **2.0**  **(0.6–5.2)** | **6.6**  **(-2.8–26.7)** | **3/0.3** | **9.0**  **(1.9–26.4)** | **26.5**  **(2.9–83.8)** |
| **Pelvic** | **0/0** | **–** | **–** | **3/0.5** | **6.6**  **(1.4–19.3)** | **22.3**  **(1.4–72.7)** | **1/0.1** | **8.1**  **(0.2–45.3)** | **23.6**  **(-2.6–147)** |
| **Table B (Continued). Standardized Incidence Ratios and Absolute Excess Risks for Soft Tissue, Genitourinary and Female Genital Subsequent Neoplasms by Calendar Year, Age at Diagnosis, Site and Follow-up** | | | | | | | | | |
| **Non-pelvic central** | **0/0** | **–** | **–** | **1/1.5** | **0.7**  **(0.0–3.7)** | **-2.6**  **(-7.6–20.8)** | **2/0.2** | **9.6**  **(1.2–34.6)** | **21.4**  **(-0.4–86.1)** |
| **Follow-up, years**  **0-5** | **1/0.1** | **11.2**  **(0.3–62.6)** | **1.8**  **(-0.1–10.7)** | **4/2.5** | **1.6**  **(0.4–4.2)** | **3.0**  **(-2.7–15.2)** | **0/0.6** | **–** | **–** |
| **5-30** | **6/0.2** | **28.6**  **(10.5–62.3)** | **6.0**  **(2.0–13.2)** | **10/5** | **2.0**  **(1.0­–3.7)** | **5.2**  **(-0.2–13.8)** | **9/1.8** | **5.0**  **(2.3–9.6)** | **17.2**  **(5.6–36.6)** |
| **>30** | **1/0.1** | **11.0**  **(0.3–61.6)** | **4.6**  **(-0.3–27.7)** | **3/4.3** | **0.7**  **(0.1–2.0)** | **-6.7**  **(-18.7–22.2)** | **1/0.8** | **1.3**  **(0.0–7.4)** | **3.1**  **(-8.9–59.5)** |
| **Abbreviations:**  **SIR=standardized incidence ratio**  **AER=absolute excess risk (mean excess subsequent primary neoplasms per 10 000 person-years)**  **CI=confidence interval** | | | | | | | | | |

| **Table C. Standardized Incidence Ratios and Absolute Excess Risks for Hematologic, Skin and CNS Subsequent Neoplasms by Calendar Year, Age at Diagnosis, Site and Follow-up** | | | | | | | | | |
| --- | --- | --- | --- | --- | --- | --- | --- | --- | --- |
|  | **Hematologic** | | | **Skin** | | | **CNS** | | |
|  | **No. Obs/exp** | **SIR (95% CI)** | **AER (95% CI)** | **No. Obs/exp** | **SIR (95% CI)** | **AER (95% CI)** | **No. Obs/exp** | **SIR (95% CI)** | **AER (95% CI)** |
| **Calendar Year**  **1958-1999** | **3/2.2** | **1.4**  **(0.3–4.0)** | **1.1**  **(-2.2–8.9)** | **7/2.7** | **2.6**  **(1.0–5.3)** | **5.8**  **(0.1–15.9)** | 6/1.1 | 5.3  (1.9–11.6) | 6.6  (1.5–16.2) |
| **1980-1999** | **4/1.6** | **2.5**  **(0.7–6.4)** | **3.5**  **(-0.8–12.6)** | **4/2.1** | **1.9**  **(0.5–5.0)** | **2.8**  **(-1.4–12.0)** | **4/0.9** | **4.7**  **(1.3–12.0)** | **4.6**  **(0.3–13.8)** |
| **2000-2015** | **3/0.6** | **5.0**  **(1.0–14.7)** | **9.1**  **(0.1–31.0)** | **2/0.9** | **2.2**  **(0.3–7.9)** | **4.1**  **(-2.6–23.9)** | **1/0.3** | **3.4**  **(0.1–18.9)** | **2.7**  **(-1.0–20.0)** |
| **Age at Diagnosis**  **0–9 years** | **2/0.2** | **10.8**  **(1.3–39.1)** | **8.5**  **(0.3–32.8)** | **1/0.2** | **6.2**  **(0.2–34.8)** | **3.9**  **(-0.6–25.2)** | **1/0.2** | **6.4**  **(0.2–35.7)** | **3.9**  **(-0.6-25.2)** |
| **10-19 years** | **3/1.1** | **2.7**  **(0.6–7.8)** | **2.2**  **(-0.6–9.2)** | **3/1.3** | **2.3**  **(0.5–6.7)** | **2.0**  **(-0.8–8.9)** | **4/0.9** | **4.7**  **(1.3–12.1)** | **3.8**  **(0.3–11.3)** |
| **≥ 20 years** | **5/3.1** | **1.7**  **(0.5–3.8)** | **3.0**  **(-2.3–13.4)** | **9/4.3** | **2.1**  **(1.0–4.0)** | **7.5**  **(-0.2–20.3)** | **6/1.3** | **4.7**  **(1.7–10.2)** | **7.4**  **(1.4–18.5)** |
| **Site**  **Extremity** | **7/3.6** | **2.0**  **(0.8–4.0)** | **2.6**  **(-0.5–8.2)** | **12/4.7** | **2.6**  **(1.3–4.5)** | **5.6**  **(1.2–5.4)** | **9/1.8** | **4.9**  **(2.3–9.4)** | **5.4**  **(1.7–11.6)** |
| **Central** | **3/0.6** | **4.7**  **(1.0–13.6)** | **7.6**  **(0.1–26.3)** | **0/0.9** | **-** | **-** | **1/0.4** | **2.7**  **(0.1–15.0)** | **2.1**  **(-1.1–17.0)** |
| **Pelvic** | **2/0.2** | **10.6**  **(1.3–38.4)** | **16.0**  **(0.5–62.1)** | **0/0.2** | **-** | **-** | **0/0.1** | **-** | **-** |
| **Table C (Continued). Standardized Incidence Ratios and Absolute Excess Risks for Hematologic, Skin and CNS Subsequent Neoplasms by Calendar Year, Age at Diagnosis, Site and Follow-up** | | | | | | | | | |
| **Non-pelvic central** | **1/0.4** | **2.2**  **(0.1–12.2)** | **2.8**  **(-2.2–26.2)** | **0/0.7** | **-** | **-** | **1/0.3** | **4.0**  **(0.1–22.3)** | **3.9**  **(-1.2–27.7)** |
| **Follow-up, years**  **0-5** | **6/1.0** | **6.1**  **(2.2–13.3)** | **9.8**  **(2.4–23.6)** | **2/1.0** | **1.9**  **(0.2–6.9)** | **1.9**  **(-1.6–12.1)** | **3/0.5** | **6.3**  **(1.3–18.3)** | **4.9**  **(0.3–16.2)** |
| **5-30** | **3/2.2** | **1.4**  **(0.3–4.0)** | **0.8**  **(-1.6–6.7)** | **6/2.8** | **2.2**  **(0.8–4.7)** | **3.3**  **(-0.6–10.6)** | **6/1.3** | **4.8**  **(1.7–10.4)** | **4.9**  **(1.0–12.2)** |
| **>30** | **1/1.2** | **0.8**  **(0.0–4.5)** | **-1.1**  **(-6.0–21.7)** | **5/1.9** | **2.6**  **(0.8–6.1)** | **15.5**  **(-1.5–49.1)** | **2/0.5** | **3.7**  **(0.4–13.4)** | **7.3**  **(-1.5–33.5)** |
| **Abbreviations:**  **SIR=standardized incidence ratio**  **AER=absolute excess risk (mean excess subsequent primary neoplasms per 10 000 person-years)**  **CI=confidence interval** | | | | | | | | | |
